# Perception, knowledge, and interest of urologic surgery: a medical student survey

**DOI:** 10.1186/s12909-019-1794-5

**Published:** 2019-09-13

**Authors:** Bristol B. Whiles, Jeffrey A. Thompson, Tomas L. Griebling, Kerri L. Thurmon

**Affiliations:** 1 Department of Urology, University of Kansas School of Medicine, 3901 Rainbow Boulevard, Mail Stop # 3016, Kansas City, KS 66160-7390 USA; 2 Department of Biostatistics, University of Kansas School of Medicine, 3901 Rainbow Boulevard, Mail Stop # 3016, Kansas City, KS 66160-7390 USA; 3 The Landon Center on Aging, University of Kansas School of Medicine, 3901 Rainbow Boulevard, Mail Stop # 3016, Kansas City, KS 66160-7390 USA

**Keywords:** Students, medical, Knowledge, Education, Perception, Urology

## Abstract

**Background:**

Although only a limited number of medical schools require a formal educational rotation in urologic surgery, urology as a medical specialty continues to attract a large number of students into the match each year. The purpose of this study was to describe medical student awareness, perception, and knowledge of urology, to determine factors influencing students’ consideration of urology as a career, and to determine if prior urology clerkship experience is associated with differences in these variables.

**Methods:**

In this cross-sectional study, medical students were electronically surveyed in 07/2016. Self-reported and question-based knowledge of urology were determined. A total of 25 factors were assessed with a five-point Likert scale to determine their influence on students’ consideration of urology as a career. Data analysis was performed using R.

**Results:**

The survey was completed by 114 students (13.5% of all medical students). A total of 11(9.65%)students had previously participated in a urology clerkship. All students reported awareness of urology; however, only 74 students (64.9%) correctly identified the training pathway and job duties of urologists. Self-perceived knowledge of urology was poor but improved with increased medical school training. Question-based assessment also demonstrated increased knowledge with advanced medical school training (27% per year; *p* < 0.01). Prior urology clerkship experience appeared to be associated with increased urologic knowledge; however, this was confounded by year in medical school training. When assessing factors impacting students’ consideration of a career in urology, ‘combination of medicine and surgery’ was the most positively influential and ‘competitiveness of the specialty’ was the most negatively influential.

**Conclusions:**

Although medical students are aware of urology as a specialty, they perceive their knowledge of urology as poor. However, knowledge of urology increases throughout medical school training. Multiple factors influence students’ consideration of urology as a career choice. Additional studies are needed to further explore how participation in a formal urology experience alters students’ perceptions and influences their consideration of urology as a career choice.

**Trial registration:**

Retrospectively registered.

## Background

Consistent with the continued decline in urologic surgery specialty education, only a limited number medical schools in the United States require a rotation in urology [1–3]. When urology program directors were surveyed, 65% noted that their medical students could graduate without ever participating in a formal clinical exposure to urology [2]. Mandatory clinical rotations in urology are currently required at less than 20% of United States medical schools, which is substantially lower than in 1956 at 99% and in 1994 at 38% [2]. Despite limited participation in a formal educational experience, urologic surgery continues to attract a large variety of students into the match each year [4]. The relationship between participation in a urology clerkship and medical student perceptions, interest, and knowledge of urology has not been well studied. Medical student perception of urology in general has been investigated in multiple other countries and cultures [5–8]. However, to our knowledge, no studies to date have investigated medical student perceptions of urology in the United States. The purpose of this study was to describe medical student awareness, perception, and knowledge of urology as well as provide an update on factors influencing students’ consideration to pursue urology as a career choice. We hypothesized that medical student awareness and knowledge of urology are both poor but improve after participation in a formal urology clerkship. We also hypothesized that participation in a urology clerkship may impact student knowledge and factors influencing students’ consideration of urology as a career choice.

## Methods

This is a cross sectional study of students at the University of Kansas School of Medicine, who were surveyed in July 2016. This study was approved by the Institutional Review Board. Our survey was administered to all medical students, regardless of their year in training, electronically via a RedCap survey. One reminder email was sent to request additional survey responses two weeks after the initial email. Survey responses were confidential. The only identifying information collected was the student’s email address; although, all data was de-identified prior to analysis. Participation in this study was completely voluntary, and no incentives were offered for completing the survey.

The survey tool utilized in our study was constructed after performing an extensive review of the educational literature for both urologic surgery as well as other surgical subspecialties. Previous studies investigating medical student awareness were used as a model for developing our tool to assess medical student awareness of urologic surgery as a specialty [9–11]. Development of this survey occurred via an iterative process. This was not a modified survey; it was developed in its entirety by the research team. Although other similar survey instruments were used to generate ideas, no other survey was directly used to create this survey. A complete, formatted copy of our survey is included in Additional file 1. This questionnaire tool has not been previously published elsewhere. A pretest of the survey was completed by all members of the study team, including two board-certified urologic surgeons.

Prior urology clerkship experience was determined via one of the study questions. A five-point Likert scale was used to assess self-perceived knowledge of urology. Formal knowledge was assessed with six multiple choice questions. The study investigators developed these questions based on the American Urological Association’s Medical Student Curriculum since no other short assessment of medical student urologic knowledge was previously available within the literature (Additional file 1). These knowledge questions were pretested by two academic urologists to establish content validity. To establish construct validity, the survey was completed other members of the research and urology department teams.

We also developed a 25-component assessment tool to investigate factors that influence students’ consideration of urology as a career choice. Factors were graded on a five-point Likert scale. The factors assessed were based on a combination of those assessed in prior studies for multiple surgical specialties [12–17].

Data analysis was completed using the R statistical environment. Differences in Likert-type data were tested individually using two-sided Fisher’s exact tests. Overall differences in Likert-type data were tested by a multivariable analysis of variance (MANOVA) for all questions with marginal significance (*p*-value < 0.1) at the individual question level. Differences in urology knowledge were tested using a Poisson-model, to determine the number of correct questions achieved by year in medical school. A *p*-value < 0.05 was considered statistically significant for all tests.

## Results

A total of 114 students (13.5% of all medical students) completed the survey. Student demographics are in Table 1. Participation in a prior urology clerkship experience was reported by 11 students (9.65%).

**Table 1:** Medical Student Demographics

	Num. of Medical Students	
	#	(%)
Total # of Students	114	(13.5%)
Gender		
Female	61	(53.5%)
Ethnicity		
Caucasian	93	(81.6%)
African American	2	(1.75%)
Other/Unknown	19	(16.7%)
Year in Medical School		
1st Year	27	(23.7%)
2nd Year	32	(28.1%)
3rd Year	22	(19.3%)
4th Year	33	(28.9%)
Prior Urology Clerkship	11	(9.65%)

We did not perform a formal analysis of non-responders or non-response bias, so we are therefore unable to account for all causes of non-response bias. Although unable to assess at the time of this study, prior exposure to urology or lack thereof may be associated with response or non-response bias to this survey. However, we performed a sub-analysis of the number of students who generally participate in a urology clerkship each year as well as the breakdown of the gender seen in each of the different medical student classes. Approximately 10% of students participate in this elective medical student rotation each year, which correlates well with the response rate of this survey. Of the respondents, approximately ½ were female. A sub-analysis of student gender for each medical student class was performed with 50–60% of respondents being female. We then compared this to the breakdown of gender for that entire class of medical students with each class having 40–50% female students, confirming responses were well distributed from the different genders across the different class groups. This supports that we likely have an adequate representation of the entire medical student population at our institution.

### Awareness of urology

All students (100%) reported awareness of urology as a specialty. Despite this, only 74 students (64.9%) correctly identified that urologists train via their own residency program. The most common misconceptions were that training consisted of a transitional year followed by a urology residency (19 students; 16.6%), an internal medicine residency followed by a urology fellowship (15 students; 13.1%), or a general surgery residency followed by a urology fellowship (6 students; 5.3%). No students (0%) reported that urology training consisted of preliminary training in Obstetrics and Gynecology. A total of 74 students (64.9%) correctly identified the job duties of a urologist including outpatient clinic, inpatient rounds, admitting patients to the hospital, and both outpatient and inpatient surgery. The single most common misconception was the lack of recognition that urologist’s play a role in admission of patients to the hospital (83 students, 72.8%).

### Knowledge: self-perceived and question-based assessment

**Fig. 1:**
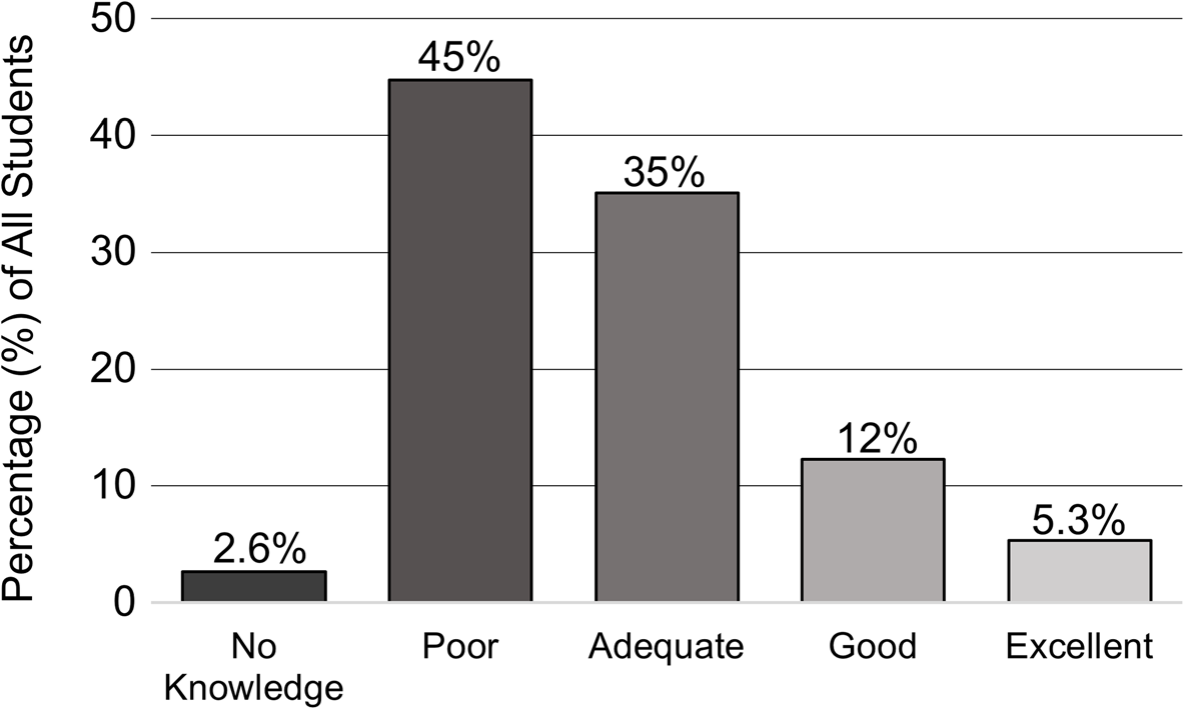
Self-perceived knowledge of urology overall

**Fig. 2:**
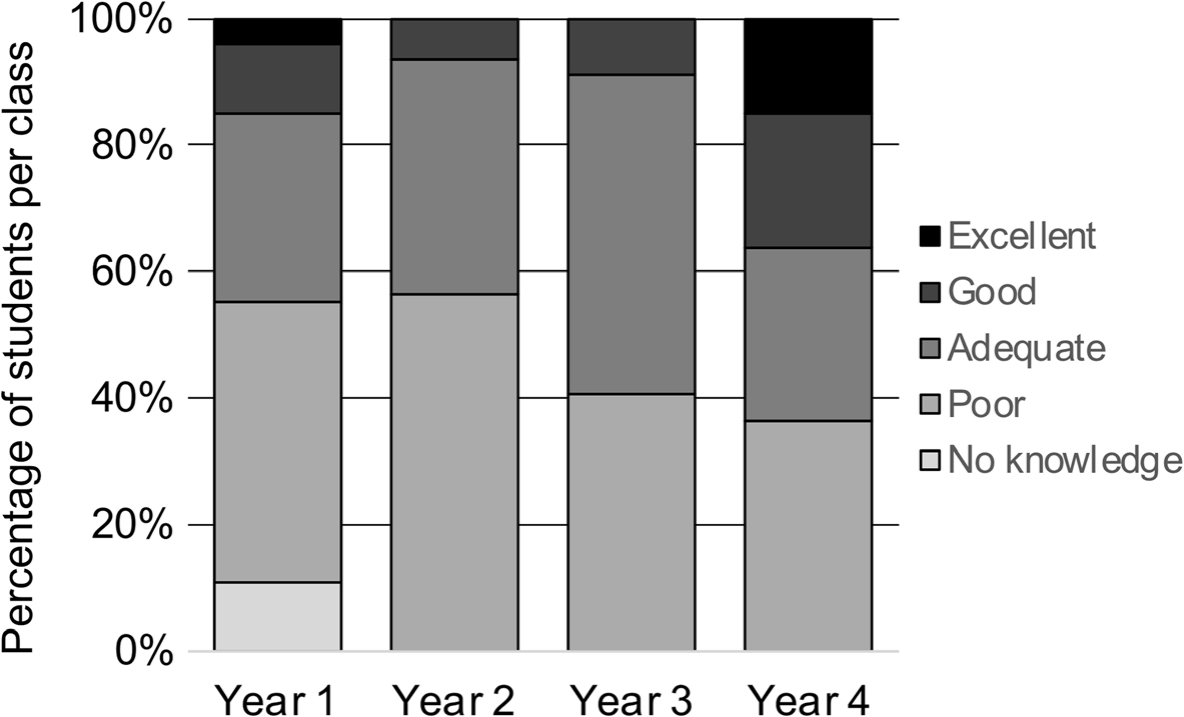
Self-perceived knowledge of urology by year in medical school training

**Fig. 3:**
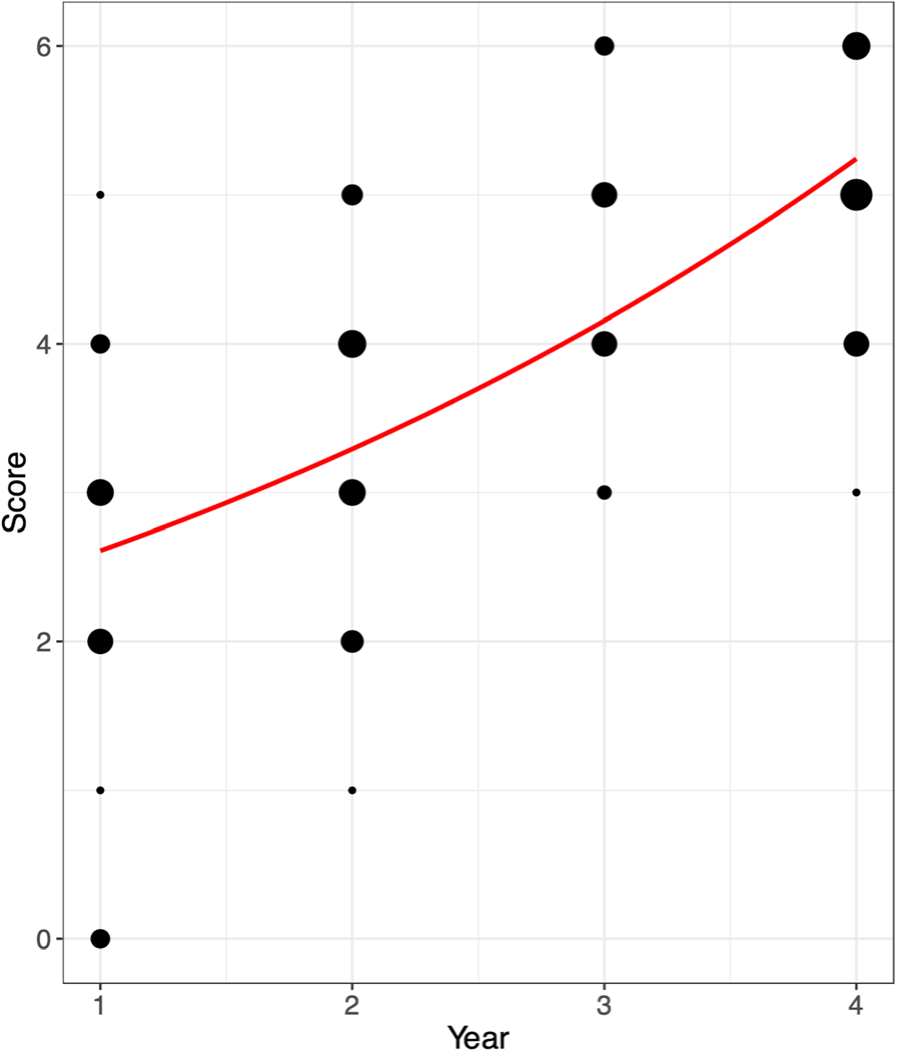
Question-based knowledge assessment of urology. The dots are scaled to represent the number of students achieving the corresponding score. The red line represents the predicted average performance under the unadjusted model (which is almost exactly the same as the adjusted model)

Self-perceived knowledge of urology compared to other clinical subjects was generally ranked by medical students as poor (Fig. 1). Prior clerkship experience as well as increased year in medical school was associated with improved self-perceived knowledge of urology (Fig. 2) (both with *p* < 0.01). However, when comparing only students eligible for prior participation in a urology clerkship (M4 students since survey administered at the beginning of an academic year), clerkship experience was no longer a significant predictor of self-perceived knowledge (*p* = 0.28). Formal urology knowledge assessment demonstrated that more advanced year in medical school was associated with increased number of knowledge questions answered correctly. We performed a Poisson regression of the number of questions answered correctly, regressed on the year of training in medical school. The model was adjusted for gender, prior clerkship, and campus. However, the only significant effect was for year of medical school (Fig. 3). We observed an average increase of about 27% in the number of knowledge questions answered correctly for each higher year of medical school training (*p* < 0.01) Without other covariates in the model, prior clerkship experience was also associated with increased number of knowledge questions answered correctly (p < 0.01); however, this was confounded by year of training in medical school.

### Influence on consideration of urology as career choice

A total of 111 students (97.3%) responded to the influence assessment questions. Of the 25 factors assessed, the most positively influential factors on student’s consideration of urology as a career choice included (students reporting positive influence via a Likert response 4 or 5): 1) ‘Combination of medicine and surgery’ (67 students; 60.3%), 2) ‘Earning potential’ (55 students; 49.5%), 3) ‘Use of innovative technology’ (53 students; 47.7%). The most negatively influential factors included (students reporting negative influence via a Likert response of 1 or 2): 1) ‘Competitiveness’ (37 students; 33.3%), 2) ‘United States Medical Licensing Exam (USMLE) Step 1 score’ (31 students; 27.9%), and 3) ‘Lack of awareness’ (25 students; 22.5%). A color-coded heat map of all 25 influence factors assessed can be found in Additional file 2.

We examined three potential explanatory factors for these influence variables: gender, amount of debt (high/low), or prior urology clerkship experience. For each, we conducted individual level tests of significance for each question, using Fisher’s exact test. Then, using any factors that were marginally significant (*p* < 0.1), we built MANOVA models, using gender, amount of debt, or prior urology clerkship as the explanatory variables. There were no detectable differences in influence factors when assessed for gender or amount of debt. However, there were with prior urology clerkship experience. A multivariate model assessing the relationship of prior clerkship to personality fit, family member in urology, USMLE Step 1 score, prestige, early match, earning potential, career opportunities, patient relationships, use of innovative technology, lifestyle after residency, family or social demands, and lack of awareness of urology were significant (*p* < 0.01). We then tested the univariate relationships (one variable at a time) and after adjusting for multiple tests found only career opportunities significant. Thus, prior urology clerkship may be associated with viewing career opportunities a positively influential factor in considering urology as a career. In fact, 91% of students with prior clerkship rated this 4 or higher, compared to just 36% of other students.

Of the 11 students who participated in a urology clerkship, none reported that their interactions with the urology residents negatively impacted their consideration of urology as a career choice. A total of nine of these students (81.8%) rated their resident interaction as a positive influence, indicated by a 4 or 5 on our Likert scale. Only four students (3.51%) were planning to pursue a career in urology. The associations between a plan to pursue a career in urology and the influence factors were unable to be assessed due to inadequate statistical power.

## Discussion

Medical students are aware of urology as a specialty but are not confident on the training or duties of urologists in general. Knowledge of urology is poor, both by self-assessment and question-based assessment but improves with advanced medical school training. When evaluating medical student knowledge, the cofounding between year in medical school and participation in a urology clerkship is consistent with the medical student training model present at our institution when the survey was administered, where only 3rd and 4th year students participate in urology clerkship experiences.

Increased year in medical school training is associated with improved self-perceived knowledge of urology. Their knowledge likely improves secondary to clinical and educational exposure to the field; although the current level of exposure is clearly still inadequate considering the overall perception of their knowledge as less adequate compared to other clinical subjects. Having seen patients with urological issues or having worked with urology providers, they most likely appreciate the vast complexity of the specialty, and assess their current level of knowledge in relation to this.

Our findings regarding factors positively influencing student consideration to pursuing urology as a career choice are similar to those described by Kerfoot, et al. [16] However, our study varied regarding the most negatively influential factors. In their study, narrowness of the specialty, unattractive lifestyle, and demands of surgical residency were the most negatively impactful while in our study these included competitiveness, USMLE Step 1 score, and gender distribution. Although various factors influence students’ consideration of urology as a career choice, additional studies are needed to determine how participation in a urology clerkship alters these factors. More detailed exploration into the role of the gender distribution of urology and its influence on student’s consideration to pursue to urologic surgery as a career is also of interest.

Similar to the majority of medical schools across the United States, urologic surgery has not been a required clerkship rotation for our students. Although our overall response rate was low at 13.5%, this study likely provides an accurate sampling and representation of this student population with approximately 10% of students having participated in a urology clerkship both in the study as well as at our institution overall.

There is concern that the current status of medical student urological education is inadequate. Although a single solution is unlikely to bridge the knowledge gap, encouraging more students to participate in urologic educational experiences are likely to be beneficial. Implementing required urology lectures in the preclinical context and required clinical rotations in later years would both provide ample opportunities to further student exposure to the field of urology. Academic medical centers have an obligation to expose students to smaller sub-specialties such as urology. And, urology is unique in this regard, since primary care and internal medicine physicians are commonly the first provider seen by patients for some common urologic issues such as benign prostatic hyperplasia or gross hematuria. The American Urological Association agrees that it is of the utmost importance that medical students learn the essentials to the work up and management of these common conditions, even if they do not elect to pursue a career in urology [18]. Medical students and future physicians are likely to feel more confident in the work up and treatment of these conditions after having participated in a formal urology clinical experience, further emphasizing the importance of increased exposure to this field.

## Conclusions

Compared to other clinical subjects, medical students perceive their knowledge of urology as poor. This is concerning since basic urology knowledge is important in the practice of the majority of medical specialties. However, their self-perceived as well as question-based knowledge of urology improves with increased medical student training. Additional studies are warranted to further explore how participation in a formal educational experience in urology alters students’ perceptions and factors influencing consideration of urology as a potential career choice as well as to identify methods to increase medical student comfort and knowledge on urologic topics. Additional research identifying the impact of medical student knowledge on non-urology practice is also of interest.

## Supplementary information

Additional file 1. Survey instrument administered to assess medical student perception, knowledge, and interest in urologic surgery.

Additional file 2. Color-coded heat map of influence factors sorted by most positive to most negatively influential.

## Data Availability

The datasets used and analyzed during the current study are available from the corresponding author on reasonable request.

## Abbreviations

MANOVA: multivariable analysis of variance
USMLE: United States Medical Licensing Exam
