# Supplementary material for: Perception, knowledge, and interest of urologic surgery: a medical student survey
Source: BMC Med Educ. 2019 Sep 13;19:351. doi: 10.1186/s12909-019-1794-5 (PMC6743171; doi:10.1186/s12909-019-1794-5)
Supplement: Supplementary file 1 — Survey instrument administered to assess medical student perception, knowledge, and interest in urologic surgery. [file 12909_2019_1794_MOESM1_ESM.pdf]

Perception, knowledge, and interest of urologic surgery: a medical student survey

Your participation in this survey is voluntary. You may choose not to take the survey or to stop responding at any time. Your completion of the survey serves as your voluntary agreement to participate in this research project.

Email address

____
(ONLY used to match subsequent surveys taken at a later date.)

Gender

- ◯ Female
- ◯ Male
- ◯ Other
- ◯ Prefer not to answer

Ethnicity

- ◯ African American
- ◯ American Indian
- ◯ Caucasian
- ◯ Hispanic
- ◯ Other
- ◯ Prefer not to answer

Year in Medical School

- ◯ 1st year
- ◯ 2nd year
- ◯ 3rd year
- ◯ 4th year
- ◯ Graduate
- ◯ Other

Did you match into a residency program this year?

- ◯ Yes
- ◯ Yes, but into a prelim year only.
- ◯ No

Please select the speciality into which you matched for residency.	◯ Anesthesiology<br>◯ Dermatology<br>◯ Diagnostic Radiology/Nuclear Medicine<br>◯ Emergency Medicine<br>◯ Family Medicine<br>◯ Internal Medicine<br>◯ Internal Medicine + Pediatrics<br>◯ Interventional Radiology<br>◯ Neurological Surgery<br>◯ Neurology<br>◯ Nuclear Medicine<br>◯ Obstetrics and Gynecology<br>◯ Ophthalmology<br>◯ Orthopaedic Surgery<br>◯ Otolaryngology<br>◯ Pathology-Anatomic and Clinical<br>◯ Pediatrics<br>◯ Physical Medicine and Rehabilitation<br>◯ Plastic Surgery<br>◯ Preventive Medicine<br>◯ Psychiatry<br>◯ Radiation Oncology<br>◯ Radiology-Diagnostic<br>◯ Surgery-General<br>◯ Thoracic Surgery-Integrated<br>◯ Urology<br>◯ Vascular Surgery-Integrated
If other year in medical school, please specify.	__________________________________
Campus location	◯ Kansas City<br>◯ Kansas City + Wichita (2+2 years)<br>◯ Wichita<br>◯ Salina
Please indicate the amount of debt you currently have.	◯ No debt<br>◯ < $50,000<br>◯ $50,000 - $99,999<br>◯ $100,000 - $149,999<br>◯ $150,000 - $249,999<br>◯ > $250,000<br>◯ Prefer not to answer<br>(Responses are kept confidential.)

At the present time, which of the following specialties is your top career choice? Please select only one.	◯ Anesthesiology<br>◯ Dermatology<br>◯ Diagnostic Radiology/Nuclear Medicine<br>◯ Emergency Medicine<br>◯ Family Medicine<br>◯ Internal Medicine<br>◯ Internal Medicine + Pediatrics<br>◯ Interventional Radiology<br>◯ Neurological Surgery<br>◯ Neurology<br>◯ Nuclear Medicine<br>◯ Obstetrics and Gynecology<br>◯ Ophthalmology<br>◯ Orthopaedic Surgery<br>◯ Otolaryngology<br>◯ Pathology-Anatomic and Clinical<br>◯ Pediatrics<br>◯ Physical Medicine and Rehabilitation<br>◯ Plastic Surgery<br>◯ Preventive Medicine<br>◯ Psychiatry<br>◯ Radiation Oncology<br>◯ Radiology-Diagnostic<br>◯ Surgery-General<br>◯ Thoracic Surgery-Integrated<br>◯ Urology<br>◯ Vascular Surgery-Integrated
Level of confidence in speciality choice listed above?	◯ Not confident<br>◯ Fairly confident<br>◯ Very confident
Have you changed speciality choice or interest since entering medical school?	◯ Yes<br>◯ No<br>◯ No, because I was unsure before now.

Urology Specific Questions

Are you aware of a speciality called Urology?	◯ Yes<br>◯ No
Define the role of a Urologist: Manages diseases involving...	☐ Female and male urinary tract<br>☐ Child urinary tract<br>☐ Female reproductive organs<br>☐ Male reproductive organs<br>☐ Proteinuria and glomerulonephritis<br>(Select all that apply.)
A urologist does the following...	☐ Has outpatient clinic<br>☐ Does ward rounds in the hospital<br>☐ Admits patients to the hospital<br>☐ Performs outpatient procedures<br>☐ Performs inpatient procedures/surgeries<br>(Select all that apply.)

A urologist is trained via the following pathway after medical school...	◯ General Surgery residency followed by urology fellowship<br>◯ Internal Medicine residency followed by urology fellowship<br>◯ Obstetrics and gynecology internship followed by urology residency<br>◯ Transitional year followed by urology residency<br>◯ Urology as its own residency program
How does your knowledge of urology compare to other clinical subjects?	◯ Excellent<br>◯ Good<br>◯ Adequate<br>◯ Poor<br>◯ No knowledge
Have you completed a clinical clerkship/rotation in Urology during your medical school training?	◯ Yes, completed by choice<br>◯ Yes, completed by requirement<br>◯ Plan to complete in future by choice<br>◯ Plan to complete in future by requirement<br>◯ No, do not plan to complete in future
Duration of your clinical rotation/clerkship in urology?	◯ 1 week<br>◯ 2 weeks<br>◯ 4 weeks<br>◯ other
When did you participate in your urology rotation/clerkship?	☐ During the first 6 months of 3rd year<br>☐ During the last 6 months of 3rd year<br>☐ As a 4th year elective<br>☐ Other
If you specified the time of your rotation/clerkship experience in urology as "other", please describe below.	______________________________
Were you considering a career in Urology prior to the clerkship experience?	◯ Yes<br>◯ No
How did the clinical rotation/clerkship in Urology influence your awareness of urology?	◯ Positive influence<br>◯ Neutral influence<br>◯ Negative influence

Please check the influence each of following on your consideration to pursue a career in Urology.

	Strongly Negative Influence	Slightly Negative Influence	Neutral Influence	Slightly Positive Influence	Strongly Positive Influence
Experience on urology clinical rotation/clerkship	◯	◯	◯	◯	◯
Interactions with urology residents	◯	◯	◯	◯	◯

Please check the influence each of following on your consideration to pursue a career in Urology.

	Strongly Negative Influence	Slightly Negative Influence	Neutral Influence	Slightly Positive Influence	Strongly Positive Influence
Coursework in medical school	○	○	○	○	○
Personality fit	○	○	○	○	○
Influenced by friends or colleagues	○	○	○	○	○
Family member in urology (if N/A, select neutral influence)	○	○	○	○	○
Self or family member with urologic problem (if N/A, select neutral influence)	○	○	○	○	○
Prior clinical exposure (shadowing or mentor)	○	○	○	○	○
USMLE Step 1 Score	○	○	○	○	○
Prestige	○	○	○	○	○
Competitiveness	○	○	○	○	○
Early match process	○	○	○	○	○
Length of training	○	○	○	○	○
Gender distribution in Urology	○	○	○	○	○
Financial earning potential	○	○	○	○	○
Career opportunities	○	○	○	○	○
Academic opportunities including research	○	○	○	○	○
Intellectual challenge	○	○	○	○	○
Patient relationships	○	○	○	○	○
Integration of medicine and surgery	○	○	○	○	○
Use of technology in urology (ex: lasers, robots)	○	○	○	○	○
Lifestyle during residency	○	○	○	○	○
Lifestyle after training	○	○	○	○	○
Family or social demands	○	○	○	○	○
Awareness of Urology	○	○	○	○	○
Other	○	○	○	○	○

If selected other factor as influential, please explain here. __________________________________

Urology Knowledge Assessment

A 32-year-old female complains of the sudden sensation to urinate multiple times per day for the past year. Work up by her primary care physician including a urine analysis is normal. What is the first line treatment for this patient?	◯ Anticholinergic-antimuscarinic medication<br>◯ Botulinum toxin injections into the bladder<br>◯ Pubovaginal sling operation<br>◯ Timed voiding every one to two hours
A 27-year-old male presents to the emergency department with acute, colicky flank pain radiating to the scrotum on the left side. He also reports hematuria. Which of the following imaging modalities is used to visualize the most likely cause of his pain?	◯ Abdominal x-ray<br>◯ CT scan<br>◯ Intravenous pyelogram<br>◯ MRI
A 55-year-old male is found to have an elevated prostate specific antigen (PSA) on routine screening by his primary care physician. The next step is which of the following?	◯ Biopsy of the prostate<br>◯ Perform digital rectal exam<br>◯ Refer to Urology<br>◯ Surgical removal of the prostate
Phosphodiesterase (PDE) inhibitors such as sildenafil are FDA approved for the treatment of erectile dysfunction. Which of the following is true about these drugs?	◯ Do not work in the majority of men<br>◯ Should be avoided in patients taking nitroglycerin<br>◯ Trigger an erection without the need for other sexual stimulation<br>◯ Work by blocking the effects of nitric oxide
A 12-week-old baby girl presents to the emergency department with a fever, poor appetite, irritability, and hematuria. Urine analysis obtained by catheterization shows WBCs, nitrites, and leukocyte esterase. What is the most likely etiology for her symptoms?	◯ Posterior urethral valves<br>◯ Ureteropelvic junction obstruction<br>◯ Urethral atresia<br>◯ Vesicoureteral reflux
A 23-year-old male presents to clinic with his wife with complaints of infertility after trying to conceive for > 1 year. He is 6ft 5in tall with a thin body habitus. Testicles are palpable, symmetric, and small. Semen analysis reveals no sperm. His wife is 25 years old and reports menstruation at 28 day intervals. She has had one normal pregnancy previously with a different partner. What is the most likely cause of their infertility?	◯ He has Cystic Fibrosis<br>◯ He has Klinefelter Syndrome<br>◯ He has Type 1 Diabetes Mellitus<br>◯ His partner has Polycystic Ovarian Syndrome
