# Supplementary figures and images for: Perception, knowledge, and interest of urologic surgery: a medical student survey
Source: BMC Med Educ. 2019 Sep 13;19:351. doi: 10.1186/s12909-019-1794-5 (PMC6743171; doi:10.1186/s12909-019-1794-5)

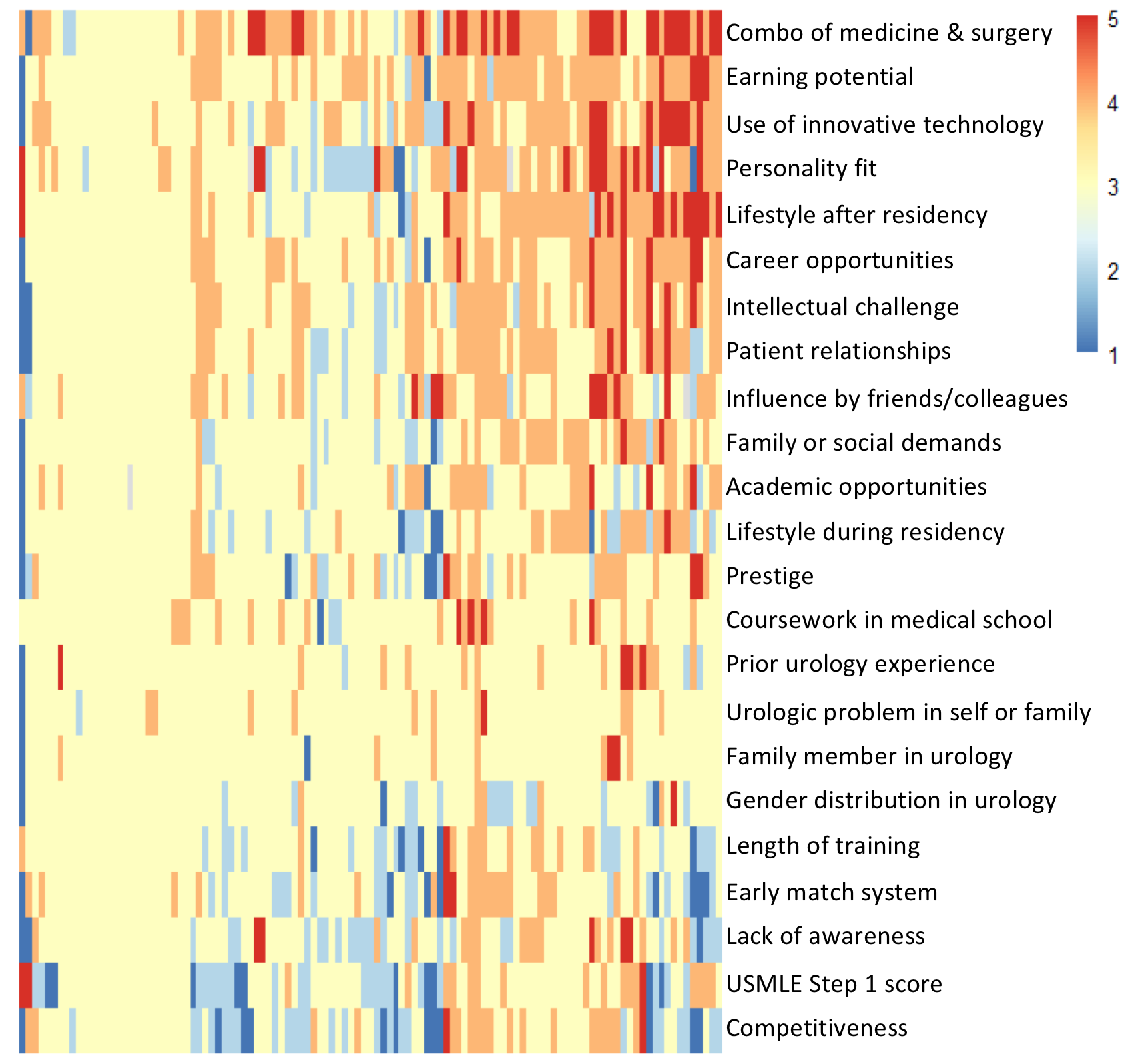

Supplement: Supplementary file 2 — Color-coded heat map of influence factors sorted by most positive to most negatively influential. [file 12909_2019_1794_MOESM2_ESM.jpg]
